# The Thermal and Mechanical Properties of Medium Chain-Length Polyhydroxyalkanoates Produced by *Pseudomonas putida* LS46 on Various Substrates

**DOI:** 10.3389/fbioe.2020.617489

**Published:** 2021-01-21

**Authors:** Christopher Dartiailh, Warren Blunt, Parveen K. Sharma, Song Liu, Nazim Cicek, David B. Levin

**Affiliations:** Department of Biosystems Engineering, University of Manitoba, Winnipeg, MB, Canada

**Keywords:** polyhydroxyalkanoates (PHAs), *Pseudomonas putida*, microbial metabolism, subunit composition, crystallinity, tensile strength, thermal properties

## Abstract

Medium chain-length polyhydroxyalkanoates (mcl-PHA) were produced by *Pseudomonas putida* LS46 cultured with a variety of carbohydrate and fatty acid substrates. The monomer compositions and molecular weights of the polymers varied greatly and was dependent on whether the substrate was metabolized via the fatty acid degradation or the *de novo* fatty acid synthesis pathways. The highest molecular weights were obtained from medium chain-length fatty acids, whereas low molecular weights were obtained from longer chain-length and more unsaturated fatty acids or carbohydrates. The differences in monomer compositions and molecular weights due to the choice of substrate did not affect the polymer thermal degradation point. The glass transition temperatures varied from −39.4°C to −52.7°C. The melting points, when observed, ranged from 43.2°C to 51.2°C. However, a profound substrate effect was observed on the crystallinity of these polymers. Reduced crystallinity was observed when the monomer compositions deviated away from C8–C10 monomer lengths. The highest crystallinity was observed from medium chain-length fatty acids, which resulted in polymers with the highest tensile strength. The polymer produced from octanoic acid exhibited the highest tensile strength of 4.3 MPa with an elongation-at-break of 162%, whereas the polymers produced from unsaturated, long-chain fatty acids remained amorphous. A comparative analysis of the substrate effect on the physical-mechanical and thermal properties of mcl-PHAs better clarifies the relationship between the monomer composition and their potential applications, and also aids to direct future PHA synthesis research toward properties of interest.

## Introduction

Polyhydroxyalkanoates (PHAs) are a diverse class of microbially-produced biopolymers known to vary in sidechain length, monomer back-bone length and functional groups (Steinbüchel and Valentin, 1995; Kim et al., 2007). The polymer structure is determined by microbial biosynthetic pathways, and is affected by the microbial species, the carbon substrate and the culturing conditions. This variability has produced PHAs with drastically different polymer properties (Brandl et al., 1988). PHAs have been compared to polypropylene, low-density polyethylene, rubber and adhesives due to the range of observed PHA properties (Anderson and Dawes, 1990; de Koning et al., 1994; Chen, 2010; Mozejko and Ciesielski, 2014). Possible applications for PHAs include molds and films for replacing single-use plastics, laminates, composites, coatings, adhesives, and biocompatible products for the biomedical (Madison and Huisman, 1999).

The commercial applications of PHAs have been limited by the comparatively high cost of production against competing petrochemical plastics. Substrate utilization has represented 30–50% of the reported PHA production costs, imparting the need for cheaper waste sources of triacylglycerides and simple sugars (Chanprateep, 2010; Jiang et al., 2016; Favaro et al., 2019). For further cost reduction, culturing conditions are optimized for high volumetric productivity of PHAs using a variety of feeding methods and co-substrates to obtain desired monomer compositions and improve the yield coefficient of carbon for PHA production (Blunt et al., 2018b).

The objective of PHA production is sustainable and renewable plastic products capable of replacing current petroleum-derived plastics that are non-biodegradable and a major source of environmental pollution. The challenge of using waste substrates to reduce PHA production costs is the affected chemical, thermal and mechanical properties of the polymer. The production and properties of medium chain-length PHA (mcl-PHA) have been studied on a wide range of substrates using various *Pseudomonas* spp. subjected to nutrient-limited minimal medium. Mcl-PHAs have been “tailor-made” based on changes in monomer composition, but better understanding of these effects on thermal and mechanical properties are required to justify a preferred monomer composition for a given application. In this study, the cell mass production, PHA production, polymer subunit composition, and molecular weights of mcl-PHAs synthesized by *Pseudomonas putida* LS46 were assessed for a suite of carbohydrate and fatty acid substrates. This analysis was conducted to further understand the effect of substrate type on mcl-PHA biosynthesis, removing the variability of strain and culturing conditions. The corresponding thermal and mechanical properties are reported. This further elucidates the biochemical response to substrate changes by these microorganisms during PHA production, and how the resulting material properties of these mcl-PHAs can direct production design for niche applications.

## Methods and Materials

### Culturing and PHA Synthesis

*P. putida* LS46 (International Depository Authority of Canada Accession Number 181110-03) (Sharma et al., 2012) was used for production of PHAs. Frozen stocks were revived using LB medium. All culturing occurred at 30°C. Minimal growth medium (Ramsay et al., 1990) was used for both inoculum and experimental conditions and adjusted to pH 7. 160 C-mM of substrate was added to minimal medium, however substrate toxicity was observed in some conditions. Hexanoic acid, heptanoic acid, octanoic acid and nonanoic acid were provided 120 C-mM (Blunt et al., 2018a), while shorter fatty acids could not be provided in sufficient concentration to effectively synthesize mcl-PHA under these conditions, and were produced with ammonium sulfate and substrate reduced to 0.1 g/L and 16 C-mM (our unpublished data). Reagent grade chemicals were used for medium production (Sigma Chemical Co., St. Louis, MO; Fisher Scientific, Toronto, ON). Food-grade vegetable oils were hydrolyzed to their respective LCFAs. The composition of canola LCFAs was 4.7% palmitic acid, 2.1% steric acid, 67.1% oleic acid, 16.8% linoleic acid, and 6.1% linolenic acid. The composition of flax LCFAs was 6.7% palmitic acid, 4.7% steric acid, 21.1% oleic acid, 15.1% linoleic acid, and 52.4% linolenic acid.

The inocula were prepared in 250 mL baffled shaker flasks with 50 mL working volumes and cultured in a shaking incubator overnight. A 1% inoculum (v/v) to 500 mL baffled experimental flasks with 150 mL working volumes were incubated in a rotary shaker for 30 h for the determination of chemical and thermal properties. Scale-up of PHA production for the determination of tensile properties was performed using 1 L baffled flasks or in a 7 L bioreactor (Applikon Biotechnology, Foster City, CA).

### Biomass Processing

The culture was centrifuged at 16,000 × g for 10 min. The supernatant was discarded, and the pellet was washed with PBS buffer before being re-suspended in H_2_O and transferred to pre-weighed aluminum dishes. The biomass was dried at 60°C for 24 h and weighed to determine cell dry mass. The PHA was converted to methyl ester monomers by methanolysis of 5 mg of dried biomass (Brandl et al., 1988). A flame ionization detector on the Agilent 7890 gas chromatograph after separation through an Agilent DB-23 column were used to detect the produced methyl esters. Response factors were determined using purchased standards where available (Blunt et al., 2018a), which were used to determine the monomer composition and PHA content. GC-MS confirmed unsaturated PHA monomers, and their response factors were assumed to be the same as their saturated counterpart. Gravimetric PHA content analysis was consistent with the PHA content determined using these response factors (data not shown). PHA extraction and purification methods were modified from Jiang et al. (2006). Extraction was performed in a soxhlet with chloroform, and twice purified by cold precipitation with methanol. ^1^H-NMR and ^13^C-NMR using a Brucker AMX-300 spectrometer (Brucker Biospin AG, Billerica, MA) were used to determine the chemical structure and monomer composition of purified PHAs. 10 mg of polymer sample was dried over Na_2_SO_4_ and re-dissolved in CDCl_3_.

### Analysis of Thermal Properties

Differential scanning calorimetry (DSC, TA Instruments Q2000, New Castle, DE) of 5–10 mg of purified PHA samples were analyzed using a heat-cool-heat protocol at a heating rate of 10 °C/min from −60°C up to 200°C. Thermogravimetric analysis (TGA) were performed as previously described (Sharma et al., 2017) from 35°C to 500°C at 5°C under air, after determining that degradation was non-oxidative using argon gas.

### Analysis of Molecular Weight

High-performance liquid chromatography (HPLC) using a Waters 1515 with Waters 2414 refractive index detector (Waters Corp., Milford, MA) using an Agilent 5 μm PLgel Mixed-C column and guard column were used to conduct gel permeation chromatography (GPC) at 30°C. A calibration curve was produced using Agilent EasiCal PS-1 polystyrene narrow molecular weight standards (Agilent Technologies, Santa Clara, CA). Purified PHA polymers were diluted to a concentration of 1.5 mg/mL in HPLC-grade chloroform and filter sterilized with 0.45 μm PTFE syringe filters (Fisher Scientific, Toronto, ON). Twenty microliter samples were injected into the HPLC-grade chloroform mobile phase with a flow rate of 1 mL/min.

### Analysis of Mechanical Properties

Tensile strips were melt-cast using a “dog-bone” stainless steel form (Precision ADM, Winnipeg, MB) in accordance with ASTM D638-03 (50 × 10 × 1 mm, LxWxT) and conditioned as per D618-13 to a relative humidity of 63%. The plates of a Lloyd LS5 tensile tester were separated at a rate of 10 mm/min.

## Results

**Figure 1:**
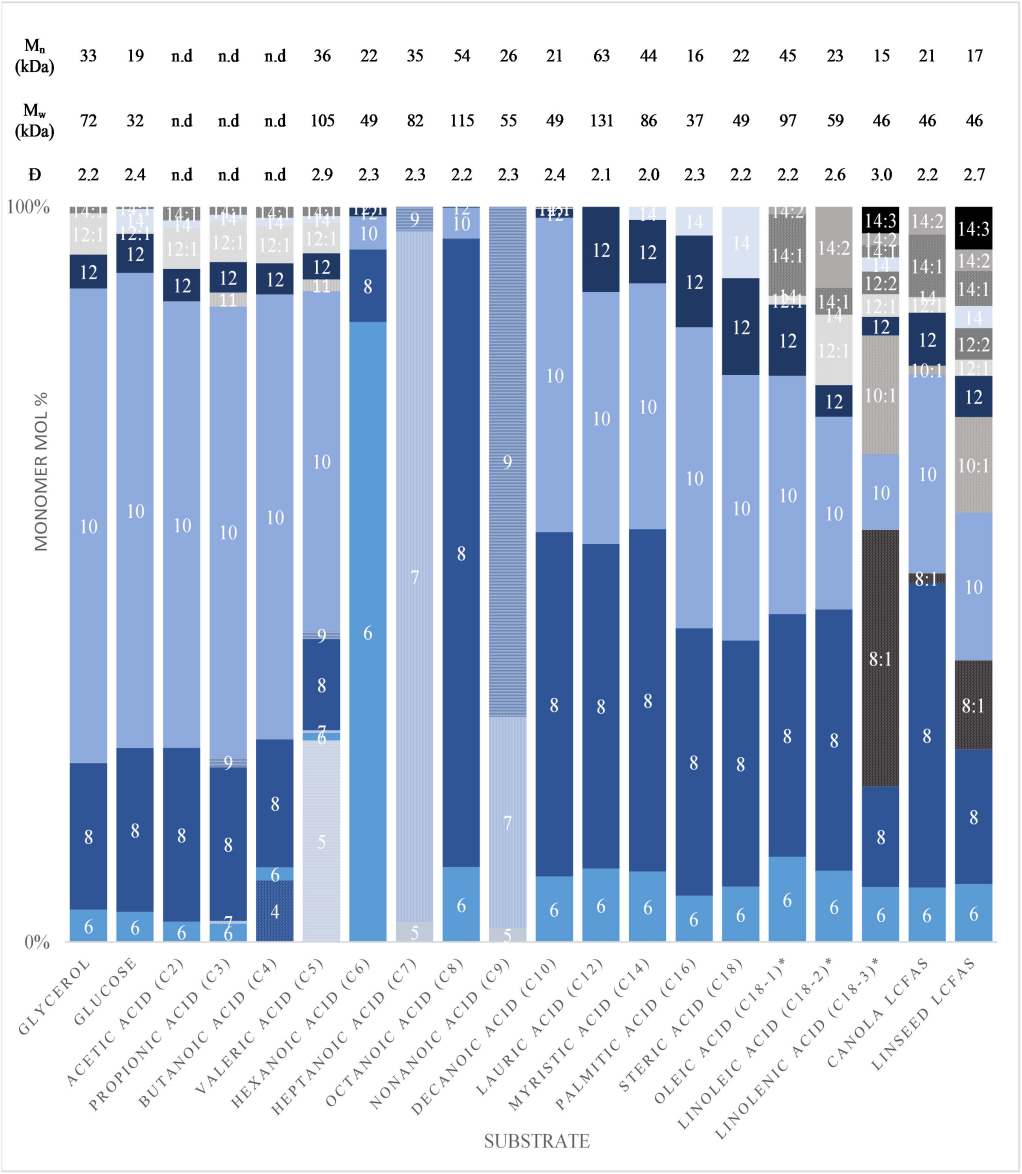
The 3-Hydroxyalkanoate monomer compositions and molecular weights produced by *P. putida* LS46 on varying carbon substrates.

Figure 1 displays the monomer compositions of mcl-PHAs produced from a variety of carbon substrates (Supplementary Table 1). Odd-numbered carbon length monomers were only observed from odd-numbered fatty acid substrates. 3-hydroxydecanoate (C10) was the dominant monomer from carbohydrates and the volatile fatty acids (VFAs) shorter than hexanoic acid. For the substrates in the range of hexanoic acid to nonanoic acid, the dominant monomer was the same length as the substrate. Even-numbered substrates of eight to eighteen carbons were all preferentially incorporated to 3-hydroxyoctanoate (C8) and C10 monomers. These results are consistent with the dominant monomers observed with *P. putida* KT2442 by which we can infer that fatty acids longer than valeric acid rely primarily upon the fatty acid β-oxidation pathway for PHA monomer production, while structurally-unrelated substrates rely solely on the fatty acid biosynthesis pathway (Huijberts et al., 1994).

Huijberts et al. (1994) suggested that the main source of longer monomers from hexanoic acid were from an elongation of hexanoic acid with acetyl-CoA, over a completely *de novo* fatty biosynthesis route. Here, the ratio of C8/C10 from hexanoic, and the inclusion of some 3-hydroxynonanoate (C9) from heptanoic acid agree with their findings. However, PHA production from propionic acid resulted in only 3.7 mol% of uneven-length monomers longer than the substrate, whereas the remaining 96.3 mol% of monomers were even-length, and longer than the substrate. A similar trend was observed by PHA production from valeric acid, except for the incorporation of 3-hydroxyvalerate (C5). PHA production from butanoic acid, with exception to the 8.5 mol% 3-hydroxybutyrate (C4) incorporated, resulted in monomer ratios similar to those obtained from glycerol and glucose. This indicates that the main route for PHA production from substrates shorter than hexanoic acid is by oxidation of the substrates to acetyl-CoA and subsequent *de novo* fatty acid biosynthesis route for PHA production.

Increased fatty acid substrate length resulted in an increased proportion of 3-hydroxydodecanoate (C12) and 3-hydroxytetradecanoate (C14). An increase in LCFA unsaturation resulted in an non-proportional increase of unsaturated 3-hydroxyacids. When fatty acid biosynthesis was required for PHA synthesis, small proportions of saturated C12 and C14 monomers along with monounsaturated 3-hydroxydodecenoic acid (C12:1) and 3-hydroxytetradecenoic acid (C14:1) were observed.

The molecular weight data corresponding to the mcl-PHAs produced by these substrates is also tabulated in Figure 1. Clear trends in PHA molecular weights based on carbon substrates could not be identified, despite the dispersity of molecular weights being consistent. Low molecular weights with higher dispersity were obtained from highly unsaturated mcl-PHAs.

The intracellular PHA content was highest from medium chain fatty acids (MCFAs) and lowest in substrates with high proportions of polyunsaturated fatty acids and steric acid, the later likely due to substrate mass transfer limitations (Table 1). The non-PHA cell mass (NPCM) ranged from 0.17 to 2.96 g/L where the lowest titers were obtained from MCFA substrates and the highest obtained from LCFAs. The higher NPCM values obtained from LCFAs could be partially explained by artificial increases due to contaminating substrate not removed during biomass processing, but these results are consistent with previous observation under microaerophilic conditions (Blunt et al., 2018a), which could impact these flask culturing conditions. PHAs produced from VFAs shorter than hexanoic acid resulted in an NPCM range of 0.25 g/L to 0.32 g/L and a PHA content range of 31.4% to 42.2%. The lower cell titer was due to one-tenth medium concentration required due to substrate toxicity.

**Table 1:** Growth and PHA production by *P. putida* LS46 on varying carbon substrates.

**Substrate**	**Biomass (g/L)**	**PHA Content (% DCW)**	**PHA Titer (g/L)**	**NPCM (g/L)**
Glycerol	3.06 ± 0.38	32.3 ± 8.9	1.01 ± 0.38	2.05 ± 0.01
Glucose	2.24 ± 0.32	20.0 ± 9.0	0.43 ± 0.16	1.81 ± 0.42
Hexanoic Acid (C_6_)	2.31 ± 0.06	69.9 ± 5.2	1.61 ± 0.10	0.70 ± 0.13
Heptanoic Acid (C_7_)	1.18 ± 0.20	74.5 ± 6.4	0.88 ± 0.18	0.30 ± 0.07
Octanoic acid (C_8_)	2.00 ± 0.69	41.8 ± 14.4	0.85 ± 0.22	1.31 ± 0.68
Nonanoic Acid (C_9_)	0.56 ± 0.06	68.7 ± 21.2	0.39 ± 0.16	0.17 ± 0.11
Decanoic Acid (C_10_)	2.52 ± 0.32	50.2 ± 10.8	1.29 ± 0.41	1.23 ± 0.10
Lauric Acid (C_12_)	2.72 ± 0.16	47.5 ± 5.3	1.29 ± 0.12	1.43 ± 0.20
Myristic Acid (C_14_)	2.55 ± 0.12	28.1 ± 6.6	0.72 ± 0.19	1.83 ± 0.17
Palmitic Acid (C_16_)	3.51 ± 0.31	41.5 ± 6.7	1.46 ± 0.29	2.05 ± 0.29
Steric Acid (C_18_)	3.13 ± 0.21	5.61 ± 2.2	0.18 ± 0.07	2.96 ± 0.20
Oleic Acid (C_18−1_)^a^	3.30 ± 0.45	46.7 ± 1.8	1.54 ± 0.27	1.76 ± 0.18
Linoleic Acid (C_18−2_)^a^	3.00 ± 0.45	49.2 ± 12.0	1.50 ± 0.56	1.50 ± 0.29
Linolenic Acid (C_18−3_)^a^	3.24 ± 0.24	16.0 ± 7.8	0.52 ± 0.25	2.72 ± 0.30
Canola LCFAs	2.85 ± 0.39	20.0 ± 1.3	0.57 ± 0.11	2.27 ± 0.27
Linseed LCFAs	2.96 ± 0.34	11.3 ± 0.8	0.34 ± 0.06	2.63 ± 0.28

a *Oleic acid, linoleic acid, and linolenic acid are 90, 60, and 70% technical grades, respectively*.

*NPCM: Non-PHA Cell Mass*.

**Figure 2:**
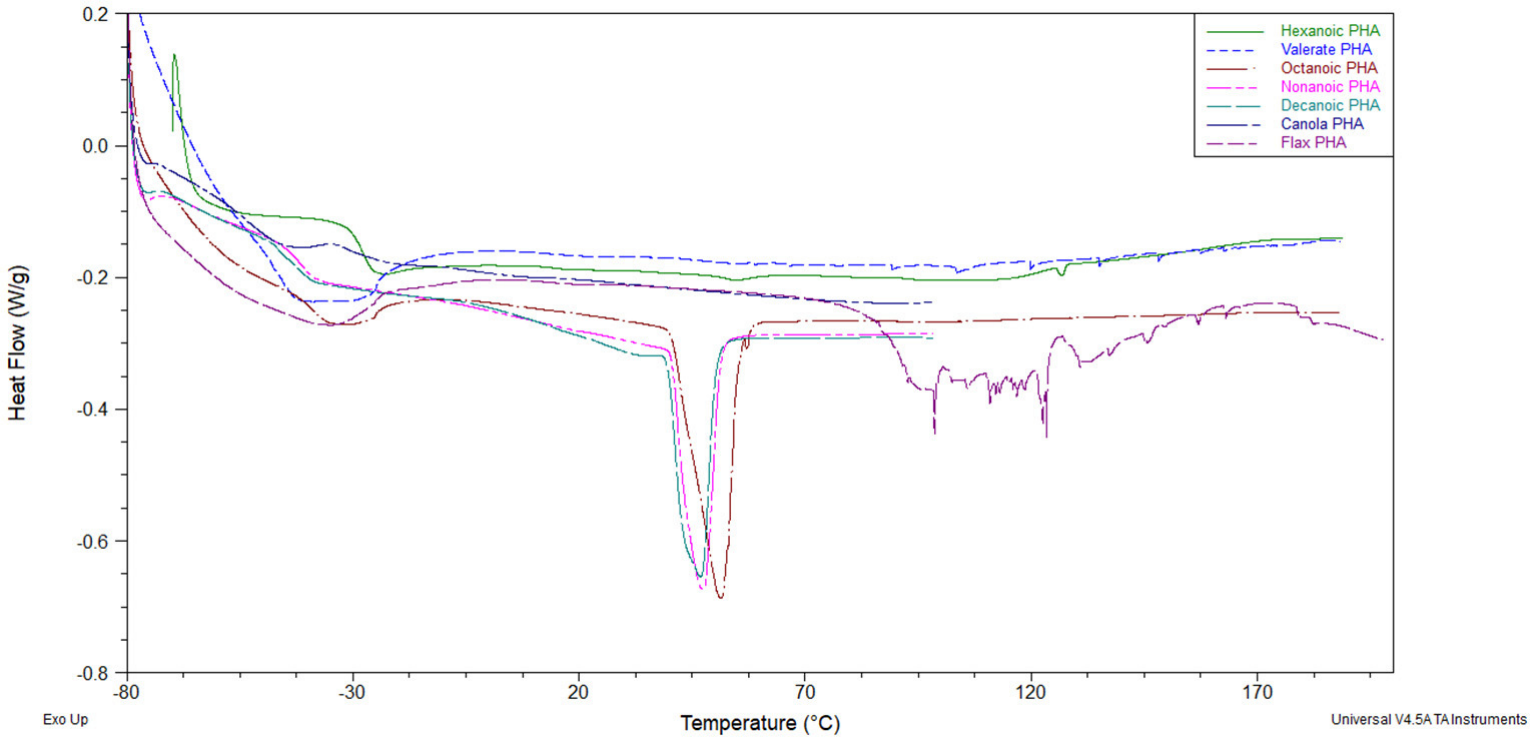
First heat thermogram overlay of mcl-PHAs obtained from various carbons substrates. Legend inset.

The crystallinity of mcl-PHAs, as determined by melt enthalpy, was highest in polymers produced from octanoic acid (Table 2). Decreases in melting temperature, glass transition temperature and melt enthalpy were observed with longer fatty acid substrates and LCFAs lacked any crystallinity, consistent with previous reports (Ashby and Foglia, 1998). Polymers produced from carbohydrates were observed to crystallize into films, but with low crystallinity and lower glass transition temperature. The melting temperature for the mcl-PHA films ranges from 43.2 to 51.2°C. Upon a cooling and second heat cycles, no melting temperature was observed, unlike PHB and PHBV polymers which observe a similar melting behavior (data not shown). This is due to the slow crystallization behavior of mcl-PHAs (Marchessault and Yu, 2005). Interestingly, polymers produced from valeric acid displayed no crystallinity, and those from hexanoic acid resulted in very low enthalpy melting at 131.1°C. The glass transition temperature of mcl-PHA from hexanoic acid and the melt enthalpies of mcl-PHA from MCFAs are distinguishable from the other mcl-PHA thermograms (Figure 2). The behavior of mcl-PHA from flax above 70°C could be the result of thermal polymer cross-linking. The tensile strength (Table 2) increased with polymer crystallinity. Mcl-PHAs produced from carbohydrates tore easily resulting in low tensile strength and low elongation-at-break. The polymers produced from octanoic, nonanoic or decanoic acid produced highly elastic polymers with elongation-at-break between 162 and 184%. Octanoic acid polymers had a significantly higher tensile strength than nonanoic or decanoic acid polymers, and lower elongation-at-break percentages. The saturated LCFAs longer than decanoic acid could not be effectively scaled-up in a bioreactor for property analysis. These fatty acids are solid at cultivation temperatures and poor mass transfer results in significant foaming issues with air sparging causing removal of the substrate.

**Table 2:** Thermal and tensile properties of mcl-PHAs.

**Substrate**	**T_**g**_ (^**°**^C)**	**T_**M**_ (^**°**^C)**	**T_**D**_ (^**°**^C)**	**ΔH_**M**_ (J/g)**	**T (MPa)**	**E (%)**	**EM (MPa)**
Glycerol	−47.0 ± 0.7	43.2 ±0.9	n.d.	10.3 ± 1.7	1.0 ± 0.1	20 ± 3.6	17.3 ± 7.2
Valerate (C_5_)	−46.9	N/A	n.d.	N/A	N/A	N/A	N/A
Hexanoic Acid (C_6_)	−29.7 ± 1.1	131.1 ± 5.6	n.d.	0.27 ± 0.23	N/A	N/A	N/A
Octanoic Acid (C_8_)	−38.1 ± 1.9	51.2 ± 0.9	249 ± 1.3	21.0 ± 0.8	4.3 ±0.4	162 ± 6.7	25.3 ± 1.8
Nonanoic Acid (C_9_)	−41.3 ± 0.9	47.2 ± 0.4	246 ± 3.1	17.6 ± 3.8	2.6 ± 0.8	184 ± 18	12.6 ± 4.7
Decanoic Acid (C_10_)	−44.0 ± 1.1	45.5 ± 1.3	254 ± 6.1	15.2 ± 0.1	2.8 ± 0.4	171 ± 1.4	10.8 0.6
Canola LCFAs	−52.7 ± 0.5	N/A	253 ± 2.3	N/A	N/A	N/A	N/A
Linseed LCFAs	−46.9 ± 5.8	N/A	n.d.	N/A	N/A	N/A	N/A

*T_g_, Glass transition temperature; T_M_, Melting Temperature; T_D_, Degradation Temperature; ΔH_M_1, Melt Enthalpy; T, Tensile Strength; E, Elongation at break; EM, Elastic Modulus; N/A, Not applicable (No melt); n.d., Not Determined*.

## Discussion

Mcl-PHA production by *Pseudomonas* spp. across a variety of substrates exhibit a biosynthetic preference for the incorporation of C8, C9, and C10 monomer lengths. *P. putida* KT442 incorporated high C10 from glycerol and glucose substrates and very small fractions of longer monomers were observed with some unsaturation. From decanoate and C18 fatty acid substrates, C8 became the dominant monomer (Eggink et al., 1992). Similar results were obtained with other *Pseudomonas* spp. across the literature (Ashby and Foglia, 1998; Haba et al., 2007; Bassas et al., 2008; Song et al., 2008; Impallomeni et al., 2011), consistent with the substrate effect on mcl-PHA monomer composition observed in this study (Figure 1). Comparing the monomer compositions of various *Pseudomonas* sp. cultivated with oleic acid, the dominant monomer varied between C8 and C10 and significant variability is seen among the other monomers, thus demonstrating the difficulty in comparing properties across the literature for the increased variability attributed to strain and culturing conditions (Eggink et al., 1992; Ashby and Foglia, 1998; Solaiman et al., 2001; Fernández et al., 2005; Conte et al., 2006; Haba et al., 2007; Impallomeni et al., 2011).

The uneven-length monomer compositions from nonanoic acid and heptanoic acid (Figure 1) are consistent with those reported by other *Pseudomonas* sp. (Thakor et al., 2005; Sun et al., 2009; Wang et al., 2011). The monomer composition obtained from growth on undecenoic acid suggested a biosynthetic preference for C9 monomers (Hartmann et al., 2006, 2010). The high monomer mol% of C6 and C7 observed from hexanoic acid and heptanoic acid, respectively, is a result of the availability of those monomers from the β-oxidation pathway and the apparent minor role of the *de novo* fatty acid biosynthesis for PHA production from fatty acids longer than valeric acid.

Unsaturated positions were conserved in the polymer sidechains of sufficient length resulting in saturated, mono- or di-unsaturated C14 monomers from petroselinic (Δ-6), oleic (Δ-9) and linoleic (Δ-9,12) acids respectively. Mono-unsaturated C12 monomers were obtained from linoleic acid. (de Waard et al., 1993). Linolenic acid (Δ-9,12,15) retains the Δ-15 unsaturation down to C8 monomers (Casini et al., 1997). Due to the added olefin and the biosynthetic preference of C8 and C10 monomers, PHAs produced from linolenic acid contain a drastically higher unsaturation content (Figure 1). The theoretical ratio of unsaturated moieties is 10.3:2.9:1 for linolenic:linoleic:oleic acids based on the average monomer lengths obtained from *P. putida* LS46 using C18 LCFA substrates; and the observed ratio was 5.3:2.5:1 which was lowered due to the technical grades of the reagents having 70, 60, and 90% purity respectively.

The molecular weights observed from *P. putida* LS46 under these conditions were smaller but comparable with literature values (Figure 1, Table 3). The molecular weight of mcl-PHAs was highest when produced from saturated fatty acids such as octanoic acid and lowest from the unsaturated LCFAs. Higher double-bond content results in lower molecular weight. Unsaturated fatty acids are hypothesized to increase polymer chain termination which resulted in smaller number average molecular weights (Ashby et al., 1998a). The molecular weights were unaffected in mcl-PHAs produced with terminal double-bonds (de Koning et al., 1994; Schmid et al., 2007), indicating that chain termination could be caused by steric effects of having kinked sidechains.

**Table 3:** Compilation of mcl-PHA average molecular weights produced from various substrates using *Pseudomonas* spp.

**PHA Polymer**	**M_**w**_ (kDa)**	**M_**n**_ (kDa)**	**Ð**	**References**
B. Carinata Oil	56	31	1.8	Impallomeni et al., 2011
Oleic Acid	57	26	2.2	Impallomeni et al., 2011
Nervonic Acid	122	63	1.9	Impallomeni et al., 2011
Erucic Acid	114	56	2.0	Impallomeni et al., 2011
Dodecanoic acid (15% C12)	100	80	1.25	Liu and Chen, 2007
Dodecanoic acid (39% C12)	*157*	108	1.45	Liu and Chen, 2007
Tetradecanoic acid (31% C14)	83	46	1.82	Liu and Chen, 2007
Tetradecanoic acid (49% C14)	95	67	1.43	Liu and Chen, 2007
Glucose + Oleic Acid	630	135	4.6	Solaiman et al., 2002
Lard	559	103	5.4	Solaiman et al., 2002
Soybean Oil	289	67	4.3	Solaiman et al., 2002
Coconut Oil	343	74	4.6	Solaiman et al., 2002
Oleic Acid	146	73	2.00	Ashby and Foglia, 1998
Tallow	142	82	1.73	Ashby and Foglia, 1998
Lard	139	84	1.66	Ashby and Foglia, 1998
Butter Oil	135	82	1.65	Ashby and Foglia, 1998
Olive Oil	119	72	1.65	Ashby and Foglia, 1998
Sunflower Oil	112	65	1.72	Ashby and Foglia, 1998
Coconut Oil	165	101	1.63	Ashby and Foglia, 1998
Soybean Oil	127	70	1.81	Ashby and Foglia, 1998
Tallow	415	147	2.8	Ashby et al., 1998a
Coconut Oil	449	133	3.38	Ashby et al., 1998b
Tallow	269	93	2.89	Ashby et al., 1998b
Soybean Oil	121	57	2.13	Ashby et al., 1998b
Octanoic acid	211.5	106^*^	1.99	Schmid et al., 2007
90% Octanoic, 10% Undecenoic	164.0	83^*^	1.98	Schmid et al., 2007
50% Octanoic, 50% Undecenoic	206.5	97^*^	2.13	Schmid et al., 2007
25% Octanoic, 75% Undecenoic	185.5	92^*^	2.02	Schmid et al., 2007
Linseed	126	60	2.1	Bassas et al., 2008
100% Octane	194	126	1.54^*^	de Koning et al., 1994
5% Octene	223	123	1.81^*^	de Koning et al., 1994
25% Octene	255	109	2.34^*^	de Koning et al., 1994
Soybean Oil/Octanoic Acid (80/20)	155	51	3.07	Hazer et al., 2009
Soybean Oil/Octanoic Acid (72/28)	162	63	2.57	Hazer et al., 2009
Soybean Oil/Octanoic Acid (50/50)	161	60	2.66	Hazer et al., 2009
Soybean Oil/Undecanoic Acid (50/50)	176	63	2.80	Hazer et al., 2009
Octanoic Acid/Undecanoic Acid (50/50)	201	64	3.11	Hazer et al., 2009
Octanoic Acid	189	51	3.69	Hazer et al., 2009
Soybean Oil	130	72	1.80	Hazer et al., 2009
Undecanoic Acid	260	135	1.92	Hazer et al., 2009

* *Author calculated based on reference data*.

*M_n_, Number-average molecular weight; M_W_, Weight-average molecular weight; Ð, Dispersity*.

Several conclusions can be postulated about the substrate effect on the thermal data presented in Table 2 when also considering the associated molecular weights and monomer compositions (Figure 1) and comparing to results across the literature (Table 4). The crystallinity of PHAs depends on the length of the monomer sidechains. The crystal structure of scl-PHA chains is disrupted by the conformational requirements of longer mcl-PHA sidechains (Marchessault et al., 1990). The incorporation of C4 and C5 monomers by native *P. putida* LS46 remains low despite using butanoic or valeric acid, instead relying on the *de novo* fatty acid synthesis pathway for mcl-PHA production. The mcl-PHA obtained from valeric acid exhibited no crystallinity in contrast to the crystallinity observed from glucose, confirming that co-polymers of scl-PHA and mcl-PHA are not isomorphic. The C6 dominant mcl-PHA produced from hexanoic acid exhibited a very weak melt enthalpy consistent with the melting temperature of PHV, whereas previous reports indicated no melt point (Marchessault et al., 1990). Homopolymers of C6 and C7 PHA monomers exhibited no crystallinity in one report using mutant *P. putida* KT2442 (Wang et al., 2011), however exhibited melting temperatures of 59 and 45°C respectively using engineered *E. coli*. The x-ray diffraction pattern of a C6 homopolymer indicated an alternative crystal structure to either scl-PHA or mcl-PHA (Abe et al., 2012). Sidechains of 3-4 carbons may be too long for crystal structures with attractive forces between parallel back-bone chains, but too short to form any potential interactions of sidechain close-packing. Alternatively, the unique crystallization of these polymers may not be isomorphic with the wild-type co-monomer composition of mcl-PHA, and the discrepancy between C6 homopolymers.

**Table 4:** Thermal properties of mcl-PHAs produced by *Pseudomonas* spp. using various feedstocks.

**PHA Polymer Feedstock**	**T_**g**_ (^**°**^C)**	**T_**m**_ (^**°**^C)**	**ΔH_**m**_ (J/g)**	**References**
Valeric Acid (100% C5)	−15.1	112.3	73.31	Wang et al., 2011
Hexanoic Acid (99% C6)	−28.2	N/A	N/A	Wang et al., 2011
Heptanoic Acid (100% C7)	−32.1	N/A	N/A	Wang et al., 2011
Octanoic Acid (96% C8)	−38.4	66.1	30.2	Wang et al., 2011
Octanoic Acid (88% C8)	−40	54	9	Jiang et al., 2012
Octanoic Acid (98% C8)	−42	62	15	Jiang et al., 2012
Nonanoic acid (70% C9)	−45	46	12	Jiang et al., 2012
Nonanoic acid (95% C9)	−48	63	27	Jiang et al., 2012
100% Octane	−29	61	23	de Koning et al., 1994
95% Octane, 5% Octene	−27	55	11	de Koning et al., 1994
75% Octane, 25% Octene	−30	N/A	0	de Koning et al., 1994
Octanoic Acid	−33.1	58.1	14.5	Schmid et al., 2007
90% Octanoic, 10% Undecenoic	−35.9	50.8	10.2	Schmid et al., 2007
50% Octanoic, 50% Undecenoic	−44.6	39.9	0.2	Schmid et al., 2007
25% Octanoic, 75% Undecenoic	−49.3	N/A	N/A	Schmid et al., 2007
Dodecanoic Acid (15% C12)	−44	53	18	Liu and Chen, 2007
Dodecanoic Acid (39% C12)	−43	65	28	Liu and Chen, 2007
Tetradecanoic Acid (31% C14)	−40	58.1	25.6	Liu and Chen, 2007
Tetradecanoic Acid (49% C14)	−40	66.8	25.1	Liu and Chen, 2007
B. Carinata Oil	−47	N/A	N/A	Impallomeni et al., 2011
Oleic Acid	−52	N/A	N/A	Impallomeni et al., 2011
Nervonic Acid	−46	50	16.1	Impallomeni et al., 2011
Erucic Acid	−43	50	15.5	Impallomeni et al., 2011
Oleic Acid	−44	42	10.7	Ashby and Foglia, 1998
Tallow	−45	44	11.4	Ashby and Foglia, 1998
Lard	−46	39	9.5	Ashby and Foglia, 1998
Butter Oil	−43	44	11.0	Ashby and Foglia, 1998
Olive Oil	−45	41	10.7	Ashby and Foglia, 1998
Sunflower Oil	−46	41	10.0	Ashby and Foglia, 1998
Coconut Oil	−38	48	12.3	Ashby and Foglia, 1998
Soybean Oil	−45	N/A	N/A	Ashby and Foglia, 1998
Linseed Oil	−51	N/A	N/A	Bassas et al., 2008

*T_g_, Glass transition temperature; T_M_, Melting Temperature; T_D_, Degradation Temperature; ΔH_M_, Melt Enthalpy*.

Monomers of C6 act as effective internal plasticizers for scl-PHA copolymers where some disruption to crystallinity can improve elastic performance (Sudesh et al., 2000). Efforts to increase the monomer mol percentage of C8 or C9 from their respective fatty acids using acrylic acid as a β-oxidation inhibitor, subsequently reducing the presence of C6 or C7 monomers, resulted in increased melting temperatures and enthalpies (Jiang et al., 2012). As previously noted, *Pseudomonas* spp. predominantly produced C8 and C10 monomers from LCFAs. Mutant *P. putida* strains were produced that incorporated higher contents of C12 and C14 monomers resulting in mcl-PHAs with increased sidechain crystallization and elevated melting temperatures (Liu and Chen, 2007; Liu et al., 2011; Abe et al., 2012). The increased content of C12 and C14 monomers in mcl-PHAs produced from glucose or unsaturated LCFAs is unlikely to contribute to their reduced crystallinity, instead the olefin moieties are responsible for the reduction in crystallinity (Ashby and Foglia, 1998). Where terminal olefins did not affect the mcl-PHA molecular weights, increased unsaturation content resulted in drastic reduction in melt enthalpy (de Koning et al., 1994; Schmid et al., 2007).

The mechanical properties of mcl-PHAs were consistent with literature reports for similar substrates (Ashby et al., 1998a; Liu and Chen, 2007; Larrañaga et al., 2014). The tensile strips always broke near the clamps due to the localization of stress from the dog-bone mold which resulted in slightly dampened results. The tensile properties are reflective of the polymer crystallinity. Mcl PHAs are viscoelastic polymers compared to thermoplastic scl-PHAs associated with the greater motional freedom of sidechain crystallinity (Marchessault and Yu, 2005; Liu et al., 2011). Improved tensile properties can be achieved by manipulating the monomer composition of mcl-PHAs to contain saturated monomers of C8 or longer. Unsaturated monomers and short-length monomers reduce the crystallinity of mcl-PHAs and detract from the viscoelastic film properties. On the other hand, the inclusion of these monomers may be preferential for alternative applications. Mcl-PHAs of low crystallinity are tacky and may find application as biodegradable adhesives (Madison and Huisman, 1999). Unsaturated moieties have also been used to improve tensile properties or produce amphiphilic polymers by co-polymer grafting, cross-linking and through chemical modification (Hazer and Steinbüchel, 2007; Kim et al., 2007).

The results described herein further elucidates the relationship between carbon substrate, the biochemical pathways of *Pseudomonas*, and the resulting polymer properties. Mcl-PHAs of varying monomer compositions were obtained from a suite of carbon substrates indicating that crystallinity, measured by melt enthalpy, was dependent on monomer length and monomer saturation which ultimately determined tensile properties. The diversity in mcl-PHA monomer composition continues to increase through the genetic modification of PHA accumulating microorganisms, culturing conditions and chemical modification of PHAs. Careful consideration of the polymer properties beyond monomer composition is required to direct culturing methods and genetic modifications toward polymers tailored for diverse applications.

## Data Availability Statement

The original contributions presented in the study are included in the article/Supplementary Materials, further inquiries can be directed to the corresponding author/s.

## Author Contributions

CD contributed to experimental design, experimental execution, data analysis, and manuscript writing. WB contributed to experimental design, laboratory assistance, and manuscript review. PS contributed *P. putida* LS46 and laboratory assistance. SL contributed laboratory infrastructure and experimental design. NC and DL contributed funding, laboratory infrastructure, intellectual input and manuscript review. All authors contributed to the article and approved the submitted version.

## Conflict of Interest

The authors declare that this study received funding from Minto BioProducts Ltd., of Minto, Manitoba Canada. This company provided both cash and in-kind contributions to the research, and its primary role in the research was to provide access to a large-scale fermentation facility for production of larger amounts of PHA polymers. The authors declare that the research was conducted in the absence of any commercial or financial relationships that could be construed as a potential conflict of interest.

## Supplementary Material

Supplementary Table 1 The 3-Hydroxyalkanoate monomer compositions produced by *P. putida* LS46 on varying carbon substrates.
