# Supplementary material for: The Thermal and Mechanical Properties of Medium Chain-Length Polyhydroxyalkanoates Produced by Pseudomonas putida LS46 on Various Substrates
Source: Front Bioeng Biotechnol. 2021 Jan 21;8:617489. doi: 10.3389/fbioe.2020.617489 (PMC7859343; doi:10.3389/fbioe.2020.617489)
Supplement: Supplementary Table 1 — The 3-Hydroxyalkanoate monomer compositions produced by P. putida LS46 on varying carbon substrates. [file Table_1.DOCX]

Supplementary Table 1. The 3-Hydroxyalkanoate monomer compositions produced by P. putida LS46 on varying carbon substrates.

| Substrate |  |  |  | |  | *Monomer Composition (mol %)* | | | | | | | | | | | |
| --- | --- | --- | --- | --- | --- | --- | --- | --- | --- | --- | --- | --- | --- | --- | --- | --- | --- |
|  | C_4_ | C_5_ | C_6_ | C_7_ | C_8_ | C_8:1_ | C_9_ | C_10_ | C_10:1_ | C_11_ | C_12_ | C_12:1_ | C_12:2_ | C_14_ | C_14:1_ | C_14:2_ | C_14:3_ |
| Glycerol | 0 | 0 | 4.4 | 0 | 19.9 | 0 | 0 | 64.5 | 0 | 0 | 4.6 | 5.6 | 0 | 0 | 0.9 | 0 | 0 |
| Glucose | 0 | 0 | 4.1 | 0 | 22.3 | 0 | 0 | 64.7 | 0 | 0 | 5.3 | 0.4 | 0 | 3.0 | 0.3 | 0 | 0 |
| Acetic Acid (C_2_)^1^ | 0 | 0 | 2.8 | 0 | 24.1 | 0 | 0 | 61.9 | 0 | 0 | 4.5 | 5.7 | 0 | 1.0 | 1.9 | 0 | 0 |
| Propionic Acid (C_3_) ^1^ | 0 | 0 | 2.5 | 0.4 | 21.1 | 0 | 1.4 | 61.9 | 0 | 1.9 | 4.2 | 5.6 | 0 | 0.9 | 1.1 | 0 | 0 |
| Butanoic Acid (C_4_) ^1^ | 8.5 | 0 | 1.8 | 0 | 17.7 | 0 | 0 | 61.4 | 0 | 0 | 4.3 | 5.2 | 0 | 1.1 | 1.5 | 0 | 0 |
| Valeric Acid (C_5_) ^1^ | 0 | 27.7 | 1.0 | 0.4 | 12.5 | 0 | 1.1 | 46.7 | 0 | 1.6 | 3.6 | 4.2 | 0 | 0.9 | 1.3 | 0 | 0 |
| Hexanoic Acid (C_6_) | 0 | 0 | 84.4 | 0 | 9.9 | 0 | 0 | 4.5 | 0 | 0 | 1.2 | 0.1 | 0 | 0 | 0 | 0 | 0 |
| Heptanoic Acid (C_7_) | 0 | 2.7 | 0 | 94.0 | 0 | 0 | 3.3 | 0 | 0 | 0 | 0 | 0 | 0 | 0 | 0 | 0 | 0 |
| Octanoic acid (C_8_) | 0 | 0 | 10.2 | 0 | 85.5 | 0 | 0 | 4.2 | 0 | 0 | 0.1 | 0 | 0 | 0 | 0 | 0 | 0 |
| Nonanoic Acid (C_9_) | 0 | 1.9 | 0 | 28.8 | 0 | 0 | 69.3 | 0 | 0 | 0 | 0 | 0 | 0 | 0 | 0 | 0 | 0 |
| Decanoic Acid (C_10_) | 0 | 0 | 8.9 | 0 | 46.8 | 0 | 0 | 42.7 | 0 | 0 | 1.2 | 0.1 | 0 | 0 | 0.2 | 0 | 0 |
| Lauric Acid (C_12_) | 0 | 0 | 10.0 | 0 | 44.1 | 0 | 0 | 34.2 | 0 | 0 | 11.6 | 0 | 0 | 0 | 0 | 0 | 0 |
| Myristic Acid (C_14_) | 0 | 0 | 9.6 | 0 | 46.6 | 0 | 0 | 33.5 | 0 | 0 | 8.6 | 0 | 0 | 1.8 | 0 | 0 | 0 |
| Palmitic Acid (C_16_) | 0 | 0 | 6.3 | 0 | 36.4 | 0 | 0 | 40.9 | 0 | 0 | 12.5 | 0 | 0 | 3.9 | 0 | 0 | 0 |
| Steric Acid (C_18_) | 0 | 0 | 7.5 | 0 | 33.5 | 0 | 0 | 36.1 | 0 | 0 | 13.2 | 0 | 0 | 9.7 | 0 | 0 | 0 |
| Oleic Acid (C_18-1_) ^2^ | 0 | 0 | 11.6 | 0 | 33.0 | 0 | 0 | 32.4 | 0 | 0 | 9.7 | 1.2 | 0 | Tr | 11.1 | 1.0 | 0 |
| Linoleic Acid (C_18-2_) ^2^ | 0 | 0 | 9.7 | 0 | 35.6 | 0 | 0 | 26.2 | 0 | 0 | 4.3 | 9.6 | 0 | Tr | 3.7 | 11.0 | 0 |
| Linolenic Acid (C_18-1_) ^2^ | 0 | 0 | 7.5 | 0 | 13.7 | 34.9 | 0 | 10.3 | 16.2 | 0 | 2.5 | 3.1 | 3.1 | 1.9 | 1.7 | 1.6 | 3.6 |
| Canola LCFAs | 0 | 0 | 7.3 | 0 | 40.8 | 1.3 | 0 | 26.7 | 1.1 | 0 | 7.1 | 2.1 | 0 | Tr | 8.4 | 3.7 | 0 |
| Linseed LCFAs | 0 | 0 | 7.9 | 0 | 18.3 | 12.1 | 0 | 20.1 | 13.0 | 0 | 5.6 | 2.2 | 4.3 | 3.0 | 4.8 | 2.9 | 5.8 |

^1^These substrates were toxic at the given concentrations, therefore both nitrogen and carbon concentrations were reduced by 90% in another unpublished work; ^2^These substrates do not represent pure fatty acids. Oleic acid, linoleic acid and linolenic acid are 90%, 60% and 70% technical grades respectively. Tr. Trace; residual linoleic acid interferes with C14 monomer peak.
